# Odontogenic Cyst with Verrucous Proliferation Exhibiting Melanin Pigmentation

**DOI:** 10.1155/2017/5079460

**Published:** 2017-03-20

**Authors:** Nidhi Manaktala, Karen Boaz, Krupa Mehta Soni, Srikant Natarajan, Junaid Ahmed, Keshava Bhat, Nandita Kottieth Pallam, Amitha Juanita Lewis

**Affiliations:** ^1^Department of Oral Pathology, Manipal College of Dental Sciences, Manipal University, Mangalore, Karnataka 575001, India; ^2^University of Colorado School of Dental Medicine, Aurora, CO, USA; ^3^Department of Oral Medicine and Radiology, Manipal College of Dental Sciences, Manipal University, Mangalore, Karnataka 575001, India; ^4^Department of Oral and Maxillofacial Surgery, Srinivas Institute of Dental Sciences, Mangalore, Karnataka 575001, India

## Abstract

Verrucous proliferation arising from odontogenic cysts is a rare entity. We report an unusual case of an infected odontogenic cyst with verrucous proliferation and melanin pigmentation in a 13-year-old male patient who presented with an intraoral swelling in relation to impacted teeth 26 and 27. The enucleated lesion was diagnosed as an odontogenic keratocyst and the patient died within two years of presentation due to multiple recurrences. The clinical, radiological, and microscopic features of the lesion are presented with an attempt to discuss the etiopathogenesis. The case hereby reported is uncommon with only eight cases reported in the literature.

## 1. Introduction

Odontogenic cysts have a diverse morphology. Though their origin and clinical presentations have been well documented, they continue to puzzle the pathologists with few rare clinicopathological presentations. Verrucous proliferation in a cystic lining is one such rarity. To the best of our knowledge only eight cases of odontogenic cysts with verrucous proliferation have been reported in English literature [1–8], of which six were reported as verrucous carcinomas arising in odontogenic cysts. We describe a case of an infected odontogenic cyst exhibiting verrucous proliferation and melanin pigmentation. It is also the first case of mortality reported among the several cases reviewed till date.

## 2. Case Report

**Figure 1:**
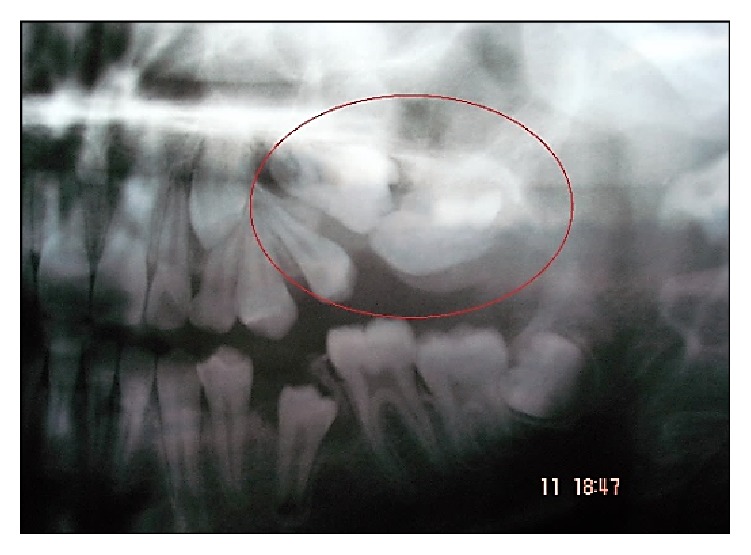
Cystic lesion (encircled) is seen surrounding the impacted 26, 27 and developing 28.

A 13-year-old male patient reported with a complaint of swelling and fetid discharge in upper left posterior tooth region since few months. On clinical examination, a solitary swelling of the left maxillary molar segment with a nonulcerated mucosa (except for a draining sinus) was evident with missing 26 and 27. The swelling was firm, had well-defined borders, and was nontender on palpation. Radiographically, a well-defined radiolucent cystic lesion surrounding impacted 26 and 27 was seen (Figure 1). Based on the age and the clinical findings, a provisional diagnosis of infected dentigerous cyst was made. The impacted teeth were surgically extracted and the cystic lining was enucleated and submitted for histopathological examination.

## 3. Gross and Histopathological Findings

**Figure 2:**
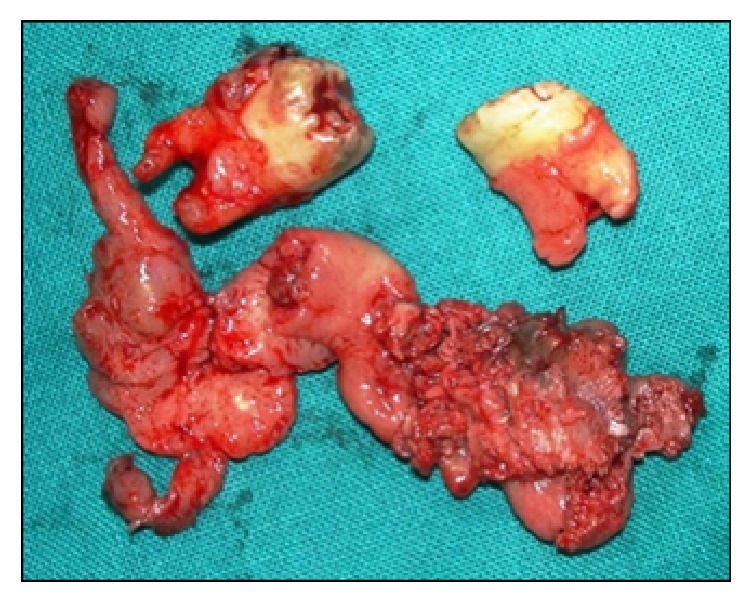
Excised specimen showing proliferative cystic lining and extracted 26 and 27.

Grossly, the excised tissue measured about 6.6 × 2.5 × 2.2 cm and the whitish-brown cystic lining showed focal areas of nodular/papillary proliferation (Figure 2).

**Figure 3:**
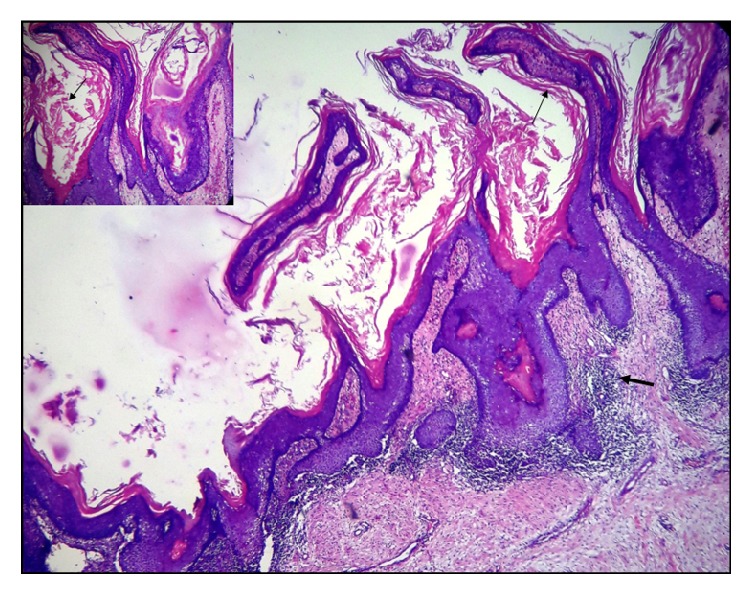
Cystic lumen lined by para- to orthokeratinised stratified squamous epithelium projecting into the lumen in the form of finger-like projections and filled with keratin. Capsule exhibiting dense inflammation (arrow) (4x, H&E).

**Figure 4:**
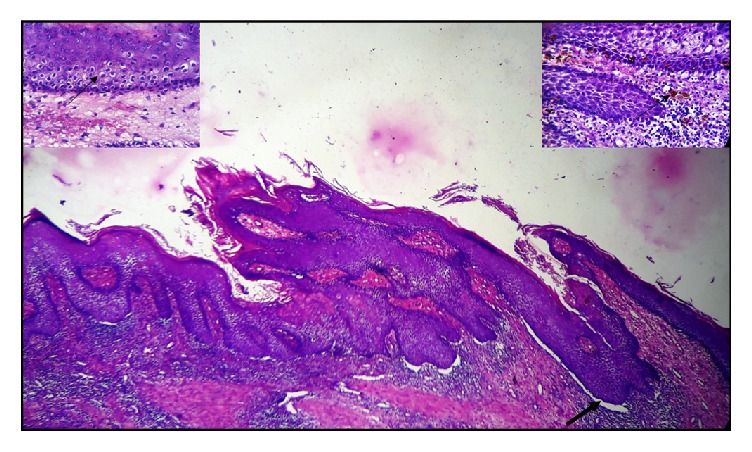
Photomicrograph showing parakeratinised stratified squamous epithelium with bulbous rete ridges lining the cystic lumen. Spinous and superficial cells exhibiting koilocytic changes (left inset); basal and suprabasal cells showing melanin pigmentation (right inset) (10x, H & E).

Microscopic examination of the specimen revealed a keratin-filled cystic lumen lined by a discontinuous five to fifteen cells thick para- to orthokeratinised epithelium (Figures 3 and 4) with keratin plugging and whorls showing (inset). The cystic epithelium showed areas of hyperplasia and broad bulbous rete ridges (Figure 4) along with areas of hypergranulosis. Koilocytic changes were also seen along with increased melanin pigmentation and melanin incontinence (inset). There was no evidence of fungal hyphae in the tissue sample as assessed by periodic acid Schiff's stain.

The supporting collagenous connective tissue capsule showed moderate to dense mixed inflammatory cell infiltrate and numerous dilated blood vessels. Many melanophages were also seen juxta-epithelially (Figure 4). Focal areas of leukocytic exocytosis were seen in the overlying epithelium.

The intraosseous location of the cyst in close association with impacted teeth suggested an odontogenic origin. The histopathological appearance was closest in compatibility to the diagnosis of an odontogenic keratocyst while not being typical of it. After due diligence and recognising the variation from classical appearances, a diagnosis of inflamed odontogenic cyst exhibiting verrucous proliferation was given. Despite the presence of a prominent verrucous component, a distinct lack of endophytic growth and “pushing” epithelial ridges precluded a diagnosis of verrucous carcinoma. Gel electrophoresis (GeneRuler ThermoFisher Scientific) confirmed the presence of human papilloma virus DNA in the tissue specimen. The lesion reportedly recurred thrice over the next two years and curettage was performed but the specimen was unavailable to us for histopathological examination at each of these recurrences. The lesion subsequently extended to the nasoethmoid region involving the orbit and perforating the base of skull anteriorly. The lesion eventually metastasized to the brain resulting in death.

## 4. Discussion

Verrucal proliferation in odontogenic lesions is an extremely rare occurrence with only a handful of cases reported in the literature till date. The demographic, clinical, radiographic, and histopathological diagnostic information of the eight reported cases of odontogenic cysts with verrucous proliferation (including verrucous carcinoma) has been compared in Table 1. These (including the present case) have been seen amongst a wide age group, ranging from 13 to 74 years. They show male preponderance (4 : 1) and are more common in the maxilla as compared to the mandible. Anterior maxilla has been reported to be affected more commonly [1–8].

Aldred et al. (2002) reported the first case of verrucous proliferation in an odontogenic cyst that occurred in a 13-year-old female patient in right maxillary alveolus, in relation to an impacted canine and the root of the lateral incisor. It was enucleated and did not recur [3]. The second case was reported by Ueeck et al. (2007) in the left mandibular ramus of a 46-year-old male with evidence of residual lesion 7 months after enucleation. Subsequent left segmental mandibulectomy and reconstruction were performed with no recurrence even after follow-up of 27 months [4]. The present case was an aggressive one as there were multiple recurrences of the lesion ultimately leading to the death of the patient.

Conventionally, a cystic lesion like an OKC/KCOT is known for its aggression but is not commonly known to metastasize. Cases of OKC have been described in the literature wherein maxillary lesions have extended to involve the orbit, skull base, and infratemporal fossa [9–11].

The occurrence of verrucous proliferation in an intraosseous cyst causes one to speculate on the supposed cause. Various etiologies for verrucous growth in odontogenic lesions proposed thus far include the presence of HPV (human papilloma virus), candidal infection, and the habit of tobacco consumption as these factors have correlated well with oral mucosal verrucous lesions [4]. However, projection of the same pathogenesis for an intraosseous odontogenic cyst is not tenable. Although tobacco plays a role in oral mucosal verrucal lesions, it is less likely to be a cause of verrucous proliferation in an intraosseous odontogenic cyst [5, 6]. Additional studies are needed to elucidate whether tobacco carcinogens in the bloodstream may circulate to chronically inflamed sites to induce verrucal proliferation [7]. While it has been suggested that candida produces nitrosamines which serve as neoplastic stimuli in verrucous lesions as stated by Ueeck et al. [4], the present case showed absence of candidal organisms in the tissue. It is therefore doubtful that candida species can be implicated in the presentation of verrucal growth in an intraosseous cyst.

Human papilloma virus (HPV) is strongly associated with oral verrucous lesions, verrucous cysts seen in skin, and even verrucous proliferation in ameloblastoma [12–14]. HPV produces the early gene proteins E6 and E7, which affect the keratinocyte cell cycle. E6 binds to the keratinocyte protein p53 (a regulating protein that inhibits keratinocyte cell division). Once p53 is bound to E6, it is degraded, resulting in an uninhibited keratinocyte mitosis and epithelial proliferation. The E7 protein binds the retinoblastoma protein (Rb) and in a similar manner disturbs the keratinocyte cell division cycle [15]. In the present case, HPV could have induced verrucous proliferation in the cystic lining as histologically there was focal koilocytic change as well as presence of the viral genome (detected in the tissue using gel electrophoresis). We speculate that the probable path for entry of the HPV in an intraosseous lesion (preexistent?) may have been via the thin lining of the maxillary sinus as the relatively thicker oral epithelium may have offered more resistance to such an invasion of the virus. Alternatively, the presence of the draining sinus might have offered a path for entry of HPV entry resulting in the verrucous proliferation. The distinctive histology of a verrucous carcinoma, namely, the endophytic growth and pushing epithelial rete ridges, was conspicuously missing. In cases of verrucous cysts of dermis, Soyer et al. agreed with the hypothesis proposed by Rous and Beard that stated that keratinocytes infected by Shope papillomavirus (SPV) can descend into the dermis and induce verrucous cysts [13]. In addition to this, Ueeck et al. emphasized the views by Kahn who had suggested that HPV in intrabony lesions may be acquired in utero or at parturition and involve the invaginating primary enamel organ [4].

Another histopathologically notable variation seen in the present case was melanin pigmentation and melanin incontinence which is unusual in an odontogenic lesion. One may surmise that the origin of the dental lamina (and further, that of the odontogenic cyst) from primitive oral mucosa may justify the presence of melanocytes [16, 17]. Neural crest cells play an important role in odontogenesis as reflected in the reciprocal induction that occurs between inner enamel epithelium and cells of the dental papilla (which originate from ectomesenchyme, a derivative of neural crest cells). Differentiation of melanocytes in this neural crest cell-rich zone is thus possible. Lawson et al. have also stated that mucosal melanocytes tend to accumulate near the attachment of dental lamina to the oral epithelium and suggested the dual role of neural crest cells in primary induction (melanocyte differentiation) as well as in the formation of tooth anlage [18]. Additionally, as most of the cases have been reported in Asian and Black patients, it is possible that the increased pigmentation may be attributed to racial variation [16].

Aldred et al. have suggested the use of the term “verrucous odontogenic cyst” [3]. However, the name “keratinising odontogenic cyst with verrucous proliferation” suggested by Ueeck et al. [4] is preferable even though it tends to veil the ambiguity of whether the lining of the odontogenic cyst undergoes verrucous proliferation or it is a “verrucous odontogenic cyst arising de novo,” which could be a new entity.

Regardless of whether such a lesion arose de novo or through transformation of a preexisting lesion, an “odontogenic cyst with verrucous proliferation” is certainly a rare entity. Of the nine cases reported with verrucous growth (including the present case) two have been reported in 13-year-old children and six were reportedly signed out as verrucous carcinoma. The patient in the present case succumbed within two years of diagnosis of the lesion due to recurrences, thus giving an insight into the aggressive behaviour of the lesion which was most likely to be due to infection by HPV. While only three of the nine cases (including present case) were assessed for presence of HPV, positivity for the HPV genome was seen only in our case (see Table 1).

The report of the present case emphasizes the need for careful enucleation and thorough histopathological examination of aggressive cystic lesions. It also warrants the need for running advanced diagnostics to identify presence of HPV infection as it might be a factor determining the prognosis of such lesions.

## 5. Conclusion

Odontogenic cysts are known to present with numerous variations, verrucous proliferation, and pigmentation being one of the rarest histological presentations. These lesions tend to be aggressive in behaviour even bearing a diagnosis of verrucous carcinoma.

The likely association of these lesions with HPV infection warrants careful and thorough investigations including PCR for detection of HPV genome and PET scans as follow-up for recurrence. Through the present report we record the ninth case of odontogenic cyst with verrucous proliferation, with a review of the previous cases in terms of histopathology and possible etiopathogenesis.

## Figures and Tables

**Table 1:** Odontogenic cystic lesions with associated verrucous lesions.

Author	Age/gender	Clinical features	Radiographic features	History of tobacco	Histopathology	Presence of HPV	Diagnosis	Treatment and follow-up
Enriquez et al. [1] (1980)	56/M	Painless mass with fistulous tract in right parotid and mandibular ramus region	Osteolytic lesion in right mandible	Present	Finger-like projections, hyperkeratosis, hyperplastic and dysplastic basal cells	HPV analysis not done	Verrucous carcinoma arising in odontogenic cyst	En bloc resection of ascending ramus

Pomatto et al. [2] (2001)	Young woman/F	Maxilla (lining of maxillary odontogenic cyst) recurrent abscesses	—	—	—	HPV negative	Verrucous carcinoma arising from a maxillary odontogenic cyst	No recurrence or metastasis after 8 months

Aldred et al. [3] (2002)	13/F	Swelling on right maxillary alveolus between impacted canine & root of lateral incisor	Radiolucency between impacted canine and lateral incisor	—	Hyperplastic epithelium with verrucous proliferation, koilocytes.	HPV negative	Odontogenic cyst with verrucous proliferation	Enucleation. No recurrence

Ueeck et al. [4] (2007)	46/M	Lesion in left posterior mandible	Radiolucency in left ramus extending till subcondylar region and coronoid process	Absent	Hyperplastic epithelium with verrucous proliferation, vacuolated cells	HPV analysis not done	Keratinizing odontogenic cyst with verrucous proliferation	Enucleation. Evidence of residual tumour after 7 months. Left segmental mandibulectomy with reconstruction plate and iliac crest bone graft. No recurrence in 27 months

Mohtasham et al. [5] (2008)	58/M	Exophytic, polypoid lesion on labial and palatal aspect of right anterior maxilla	Well-defined radiolucency	Absent	Finger-like projections, bulbous, thickened, downward growth of rete ridges with mild atypia and parakeratin plugging	HPV analysis not done	Intraosseous verrucous carcinoma originating in odontogenic cyst	Enucleation. No evidence of recurrence/metastasis after 2 years of follow-up

Dalirsani et al. [6] (2015)	49/M	Left mandibular alveolar area		—	Cauliflower like projection of epithelium along with neoplastic proliferation of odontogenic epithelium	HPV analysis not done	Verrucous carcinoma in addition to cystic ameloblastoma	Excision with preservation of rim of the inferior border. Iliac crest bone graft done to repair defects. No evidence of recurrence in 2 years of follow-up

Peng et al. [7] (2015)	74/M	Left mandible Swelling and recurrent pus discharge	Impacted 34 and a large well-defined, radiolucent lesion surrounding the crown of 34.	Present	Hyperparakeratotic stratified squamous cyst lining epithelium and downgrowth of broad and bulbous epithelial ridges with pushing-border invasion into the fibrous cystic wall	HPV analysis not done	Intraosseous verrucous carcinoma arising from an infected odontogenic cyst	Surgical excision with 5 months of follow-up. No recurrence or metastasis

Kamarthi et al. [8]	64/M	Painful swelling in left maxillary alveolus	Unilocular, radiolucent lesion extending from 21 to 25	Present	Hyperplasia and verrucous proliferation in an odontogenic cystic lining	HPV analysis not done	Intraosseous verrucous carcinoma arising from an orthokeratinised odontogenic keratocyst	Enucleation of the cystic lining. No signs of recurrence after 6 months of recall visit

Present case	13/M	Smooth, firm swelling in left posterior maxilla, in relation to impacted 26, 27	Well-defined radiolucency around impacted 26 and 27	Absent	Hyperplastic epithelium, parakeratin whorls, koilocytes, melanin pigmentation	HPV positive	Infected odontogenic cyst with verrucous proliferation exhibiting melanin pigmentation	Enucleation. Recurrence of the lesion (thrice) and death in 2-year follow-up period
